# The impact of circulating regulatory T cells number and their subpopulations on risk of lymph node metastasis

**DOI:** 10.1186/2051-1426-3-S2-P279

**Published:** 2015-11-04

**Authors:** Jerzy Wydmanski, Agata Chwieduk, Agnieszka Gdowicz-Klosok, Magdalena Glowala-Kosinska, Tomasz Latusek, Agnieszka Namysl-Kaletka, Malgorzata Kraszkiewicz, Andrzej Tukiendorf

**Affiliations:** 1 Department of Radiotherapy, Maria Sklodowska-Curie Memorial Cancer Center and Institute of Oncology, Gliwice, Poland; 2 Department of Bone Marrow Transplantation, Maria Sklodowska-Curie Memorial Cancer Center and Institute of Oncology, Gliwice Branch, Gliwice, Poland; 3 Center for Translational Research and Molecular Biology of Cancer, Maria Sklodowska—Curie Memorial Cancer Center and Institute of Oncology, Gliwice Branch, Gliwice, Poland; 4 Department of Epidemiology Maria Sklodowska-Curie Memorial Cancer Center and Institute of Oncology, Gliwice Branch, Gliwice, Poland

## Background

Regulatory T cells (Tregs) suppress the induction of immune response to cancer cells. An increased number of Tregs have been observed in many solid tumors. The aim of this study was to evaluate whether the number and percentage of circulating Tregs and their subpopulations differ in patients with gastric cancer (GC) and normal controls (NC). The relationship between Tregs subpopulations and clinical factors was analyzed.

## Materials and methods

Tregs were analyzed in 40 patients with GC and in 17 NC. Tregs were detected by multicolor flow cytometry based on CD4^+^CD25^high^FoxP3^+^ phenotype. The expressions of CD45RA (naive Tregs), HLA-DR (marker of activation), CD62L (L-selectin) and CD39 (marker of homing to inflamed regions) were investigated. The data was analyzed with STATISTICA 10 software by Mann–Whitney U test and Spearman' correlation test.

## Results

The percentages of circulating Tregs were similar in patients with GC and in NC (6.1% vs 5.2%, P>0.1), but the mean absolute number of Tregs was higher in NC compared to patients with GC (47 cells/µl vs 32 cells/µl, P < 0.1).

In addition, the frequency and the absolute number of Treg positive for CD45RA, HLA-DR and CD62L were greater in NC than in GC patients (P < 0.001). There were no correlations between the percentages of Tregs and clinical factors. However, the absolute number of Tregs (r=0.34) and their subpopulations expressing HLA-DR (r=0.32), CD62L (r=0.4) and CD39 (r=0.33) significantly correlated with higher risk of lymph nodes metastasis.

## Conclusions

Subpopulations of Tregs were differed between GC patients and NC. Surprisingly, percentages of Tregs expressing CD45RA, HLA-DR and CD62L were lower in GC patients. However, we observed correlation between the absolute number of Tregs subpopulations and disease stage. Further investigation should be taken to assess the prognostic value of Tregs subpopulations.

This study was supported by The National Science Centre of Poland (grant no. NN 403 238 140).

